# Genomics and phenomics enabled prebreeding improved early-season chilling tolerance in Sorghum

**DOI:** 10.1093/g3journal/jkad116

**Published:** 2023-05-26

**Authors:** Sandeep Marla, Terry Felderhoff, Chad Hayes, Ramasamy Perumal, Xu Wang, Jesse Poland, Geoffrey P Morris

**Affiliations:** Department of Agronomy, Kansas State University, Manhattan, KS 66506, USA; Department of Agronomy, Kansas State University, Manhattan, KS 66506, USA; USDA-ARS, Plant Stress & Germplasm Development Unit, Cropping Systems Research Laboratory, Lubbock, TX 79415, USA; Western Kansas Agricultural Research Center, Kansas State University, Hays, KS 67601, USA; Department of Plant Pathology, Kansas State University, Manhattan, KS 66506, USA; Department of Agricultural and Biological Engineering, University of Florida, IFAS Gulf Coast Research and Education Center, Wimauma, FL 33598, USA; Department of Plant Pathology, Kansas State University, Manhattan, KS 66506, USA; Center for Desert Agriculture, King Abdullah University of Science and Technology, Thuwal 23955-6900, Kingdom of Saudi Arabia; Department of Agronomy, Kansas State University, Manhattan, KS 66506, USA; Department of Soil and Crop Sciences, Colorado State University, Fort Collins, CO 80523, USA

**Keywords:** marker-assisted selection, QTL, population genomics, abiotic stress, adaptive trait, field phenotyping, KASP, molecular marker, plant genetics and genomics

## Abstract

In temperate climates, earlier planting of tropical-origin crops can provide longer growing seasons, reduce water loss, suppress weeds, and escape post-flowering drought stress. However, chilling sensitivity of sorghum, a tropical-origin cereal crop, limits early planting, and over 50 years of conventional breeding has been stymied by coinheritance of chilling tolerance (CT) loci with undesirable tannin and dwarfing alleles. In this study, phenomics and genomics-enabled approaches were used for prebreeding of sorghum early-season CT. Uncrewed aircraft systems (UAS) high-throughput phenotyping platform tested for improving scalability showed moderate correlation between manual and UAS phenotyping. UAS normalized difference vegetation index values from the chilling nested association mapping population detected CT quantitative trait locus (QTL) that colocalized with manual phenotyping CT QTL. Two of the 4 first-generation Kompetitive Allele Specific PCR (KASP) molecular markers, generated using the peak QTL single nucleotide polymorphisms (SNPs), failed to function in an independent breeding program as the CT allele was common in diverse breeding lines. Population genomic fixation index analysis identified SNP CT alleles that were globally rare but common to the CT donors. Second-generation markers, generated using population genomics, were successful in tracking the donor CT allele in diverse breeding lines from 2 independent sorghum breeding programs. Marker-assisted breeding, effective in introgressing CT allele from Chinese sorghums into chilling-sensitive US elite sorghums, improved early-planted seedling performance ratings in lines with CT alleles by up to 13–24% compared to the negative control under natural chilling stress. These findings directly demonstrate the effectiveness of high-throughput phenotyping and population genomics in molecular breeding of complex adaptive traits.

## Introduction

Climate stress is a major limiting factor for crop production. To minimize the yield losses by adverse weather patterns for providing global food security, breeding food crops with abiotic stress tolerance is critical (Mickelbart *et al*. 2015). Conventional breeding efforts to increase climate resilience in food crops were slow due to the complex genetic architecture of abiotic stress tolerance (Collins *et al*. 2008; Bernardo 2016). Further complicating conventional breeding for abiotic stress tolerance are spatiotemporal variability of environmental conditions in targeted environments between years, lack of reliable phenotyping for abiotic stress tolerance traits, and poor scalability of field trials due to limited phenotyping resources (Hickey *et al*. 2019; Varshney *et al*. 2021). Given that conventional breeding was not effective in developing abiotic stress tolerance, there is a need to utilize modern breeding tools in combination with modern phenotyping platforms in abiotic stress tolerance breeding.

Substantial advances in next-generation sequencing technologies over the past 2 decades established genomics-assisted breeding as an alternate solution to conventional breeding for complex traits (Sasaki 2005; Schnable *et al*. 2009; Abberton *et al*. 2016; McCormick *et al*. 2018; Tao *et al*. 2019; Varshney *et al*. 2021). Despite the advances in genomics-assisted breeding, breeding for complex traits mostly failed in public breeding due to weak association between the phenotype and the genomic region (i.e. trait-to-quantitative trait locus (QTL) correlation), the QTL and the marker used for selecting the trait (i.e. the QTL-to-marker correlation), and failure of QTL to function in diverse genetic backgrounds (Cobb *et al*. 2018). Increasing mapping power and resolution with multiparental populations and high density markers can improve trait-to-QTL associations for adaptive traits (Yu *et al*. 2008; Bouchet *et al*. 2017; Gage *et al*. 2020). To improve QTL-to-marker associations across diverse genetic backgrounds in a breeding program, population genomics approaches can be used to identify fixed alleles in locally adapted germplasm, that are in linkage disequilibrium (LD) with the trait of interest (Muleta *et al*. 2019). Therefore, there is a need for public breeding programs to integrate high-resolution mapping and population genomics approaches to develop abiotic stress tolerance in food crops.

Sorghum (*Sorghum bicolor*) is a tropical-origin C4 cereal crop that is sensitive to chilling temperatures (0–15°C) (Taylor and Rowley 1971; Lyons 1973). Developing chilling-tolerant sorghum hybrids could enable early-planting in temperate regions, increase growing period by 20%, reduce evaporation during fallow periods, reduce risk of terminal droughts, and increase 7–11% grain yield (Raymundo *et al*. 2021). Chinese sorghums, introduced from Africa into China ∼800 years ago and adapted to chilling temperatures, were a good source for chilling tolerance (CT) breeding (Stickler *et al*. 1962; Salas Fernandez *et al.* 2014; Marla *et al*. 2019). Coarse resolution mapping, fewer recombinant inbred lines (RILs), and low marker density, with US chilling-sensitive and Chinese chilling-tolerant biparental families, identified CT loci linked with undesirable tannin and plant height alleles (Knoll *et al*. 2007; Burow *et al*. 2010; Ostmeyer *et al*. 2020). High-resolution CT mapping with a chilling nested association mapping (NAM) population detected precise colocalization of CT alleles with undesirable grain tannin alleles at *Tan1* (*Tannin1*) and *Tan2* (*Tannin2*) genes and tall plant alleles at *Dw1* (*Dwarf1*) and *Dw3* (*Dwarf3*) genes (Marla *et al*. 2019). Coinheritance of CT loci with undesirable tannin and dwarfing alleles combined likely stymied conventional CT breeding for over 50 years.

New genome-to-phenome (G2P) approaches offer potential solutions for complex trait breeding that was hindered by low-resolution mapping, poor scalability, and complex phenotyping for abiotic stress tolerance (Cooper *et al*. 2014; Kole *et al*. 2015; Abberton *et al*. 2016; Varshney *et al*. 2021). We hypothesized G2P-enabled strong trait-to-QTL and QTL-to-marker association improves sorghum early-season CT. Based on this hypothesis, we predicted elite US sorghum lines with CT alleles, generated using marker-assisted breeding, will exhibit higher early-season seedling emergence count (EC) and seedling vigor (SV) ratings compared to the negative controls. To test our hypothesis, we developed 3 specific objectives: (1) generate molecular markers, using population genomics, to accurately detect CT alleles in diverse sorghum lines, (2) validate the ability of marker-assisted breeding to improve sorghum CT in different public sorghum breeding programs, and (3) determine if CT marker-assisted breeding improves early-season CT in sorghum hybrids. Joint linkage mapping (JLM) of Uncrewed aircraft systems (UAS) phenomic data on the chilling NAM population and fixation index (*F*_ST_) population genomic analysis generated Kompetitive Allele Specific PCR (KASP) genotyping markers for precisely introgressing CT alleles from Chinese lines into chilling-sensitive US elite lines. Segregating families and sorghum hybrids carrying CT alleles, generated using marker-assisted breeding, contained higher seedling performance ratings under natural chilling stress. Overall, the study demonstrates the power of combining phenomics and genomics-enabled prebreeding for complex adaptive traits.

## Materials and methods

### Chilling NAM population

The chilling NAM population of 771 RILs was generated by crossing the US sorghum reference line BTx623 with 3 chilling-tolerant Chinese founders, Niu Sheng Zui (NSZ; PI 568016), Hong Ke Zi (HKZ; PI 567946), and Kaoliang (Kao; PI 562744) (Marla *et al*. 2019). The chilling NAM population development, genotyping-by-sequencing (GBS), and single nucleotide polymorphism (SNP) variant calling was explained in more detail in Marla *et al*. (2019). The chilling NAM GBS data combined with previously published *Ape*KI GBS data from ∼10,000 diverse lines (Hu *et al*. 2019) was aligned to the BTx623 reference genome v3.1 (McCormick *et al*. 2018). SNP calling using Tassel 5.0 GBS v2 pipeline (Glaubitz *et al*. 2014) retained 43,320 SNPs and 750 RILs for JLM. Selected chilling NAM RILs (15FS005_NSZ, 14FS205_Kao, 14FS125_Kao, 15FS083_NSZ, 15FS032_NSZ, 15FS679_HKZ, 15FS698_HKZ, and 14FS273_Kao) with desirable agronomics and different combinations of CT alleles were used in marker-assisted CT pre-breeding.

### Early-planted field trials

The chilling NAM population was planted under natural chilling stress in 6 early- and 2 normal-planted field trials in 2016, 2017, and 2018 in Kansas, as described in Marla *et al*., (2019). Each field trial contained 2 replicates of the NAM population. Early-planted field trials were sown in April or early May, 30–45 days earlier than normal sorghum planting in Kansas (Grain Sorghum Production Handbook 1998). Manual phenotyping for SV rating was conducted in all 8 field trials. UAS phenotyping was conducted in 2 field trials, AB17 (Ashland Bottoms 2017, 39.14N, −96.63W) and MN17 (Manhattan 2017, 39.21N, −96.60W).

Six F_3_ families, generated by crossing 2 chilling NAM RILs with 3 lines from the Western Kansas Agricultural Research Center (WKARC, Hays, Kansas) sorghum breeding program, were screened for their early-season chilling response in HA19 (Hays 2019, 38.84N, −99.34W). Five replicates of each WKARC F_3_ family, completely randomized within each replication block, were early-planted on April 17. CT hybrids, from the Kansas State University (KSU) sorghum genetics and molecular breeding program (Manhattan, Kansas), were early-planted in 2019 at 2 locations: Hays and Manhattan. Each field trial consisted of 3 replicates of the parents, inbred near-isogenic lines (NILs), and hybrids with Chr2+ or Chr4+ CT allele or without CT allele (−/− sib). CT hybrid evaluations were early-planted on April 19 in HA19 and April 17 in MN19 field trials. In this study, any drop in air temperature below 15°C was considered as a chilling stress event. Air temperatures for all the early-planted field trials during planting and emergence, and at the seedling stage, were included (Supplementary Fig. 1).

### Manual field phenotyping

In natural chilling stress field trials, seedling performance traits, EC and 3 SV ratings (SV1–3), were scored on a 1–5 scale (1 for low and 5 for high) as described previously (Marla *et al*. 2019). The previous SV ratings scale (1 for high and 5 for low SV) (Maiti *et al*. 1981; Knoll *et al*. 2007; Burow *et al*. 2010) were modified by Marla *et al*. (2019) to maintain consistency with EC rating. One-week after emergence, EC was determined by counting the number of seedlings that emerged and converted to a scale of 1–5 representing 20, 40, 60, 80, and 100% emergence, respectively. Three SV1–3 ratings, scored independently of EC, were collected at weeks 1, 2, and 4, respectively, after emergence. SV ratings were scored on a rating scale of 1–5 with a rating of 1 for low and 5 for robust vigor. In the HA19 field trial, SV1–3 ratings were collected at weeks 1, 2, and 4 after emergence. In MN19 and AB19 field trials, EC and SV1 ratings were collected at week 1 after emergence, and SV2 ratings at week 2 after emergence.

### High-throughput phenotyping with UAS and data processing

UAS high-throughput phenotyping (HTP) was conducted on AB17 and MN17 early-planted field trials. HTP data in the 2017 chilling NAM trials were collected by a DJI Matrice 100 quadcopter (DJI, Shenzhen, China) carrying a MicaSense RedEdge-M multispectral camera (MicaSense Inc., USA). HTP data were collected throughout the entire growth cycle but only early-stage data, up to 45 days after emergence, were analyzed in this study. Flight plans were created using CSIRO mission planner application and missions were executed using the Litchi Mobile App (VC Technology Ltd., UK, https://uavmissionplanner.netlify.app/). Aerial image overlap rate between 2 geospatially adjacent images was set to 80% both sequentially and laterally to ensure optimal orthomosaic photo stitching quality. All UAS flights were set at 20 m above ground level at 2 m/s and conducted within 2 h of solar noon. To improve the geospatial accuracy of orthomosaic images, white square tiles with a dimension of 0.30 m × 0.30 m were used as ground control points and were placed at 4 corners of the field before image acquisition and surveyed to centimeter-level resolution using the Emlid REACH RS + Real-Time Kinematic Global Navigation Satellite System unit (Emlid Ltd, Hong Kong, China). A semi-automated image processing pipeline developed by Wang *et al*., (2020) was used to generate field orthomosaic photos and extract plot-level normalized difference vegetation index (NDVI) (Rouse *et al.* 1974) and crop coverage.

### Statistical analyses

Correlation between AB17/MN17 SV ratings and UAS NDVI data was determined using averaged values of 2 replicates from each field trial. Pearson pairwise correlation analysis was performed using the *cor* function in R package. Chi-square test (χ^2^), using R *chisq.test*, was conducted to determine whether there was a significant difference between observed and expected frequencies of CT vs chilling-susceptible (CS) alleles from KASP genotyping in intercross populations generated from the Chinese and US elite parents. In the WKARC breeding program F_3_ families, statistical comparisons were conducted between the control F_3_ family without any CT alleles (−/− sib) and individual F_3_ families with different CT allele combinations using a Dunnett's test. Boxplots were generated using ggplot2 R package (Wickham 2016). To determine if chromosome 2 or 4 (Chr2+ or Chr4+) CT alleles improved seedling performance traits, EC, SV1, and SV2, in CT hybrids, statistical comparisons were conducted between the US and Chinese parents, NILs with Chr2+ or Chr4+ or −/− sib, and hybrids with Chr2+ or Chr4+ or −/− sib using a Dunnett's test.

### Joint linkage mapping

GBS of the chilling NAM population generated 43 K SNPs for JLM analysis. JLM was performed individually for each location with the averaged data of 2 replicates. JLM of AB17 and MN17 NDVI values was conducted, as previously described (Marla *et al*. 2019), using the stepwise regression approach in TASSEL 5.0 (Glaubitz *et al*. 2014). Entry and exit limit of forward and backward stepwise regressions was 0.001 and threshold cut off was set based on 1000 permutations. Allelic effects of individual QTL were expressed relative to the BTx623 allele, where alleles with positive- or negative-additive effects were derived from BTx623 or Chinese founders, respectively.

### Population genomics analysis

A collection of 30 Chinese (Supplementary Table 1) and 390 sorghum association panel (SAP) lines, a subset of the 10 K GBS sorghum lines (Hu *et al*. 2019), were used to calculate the fixation index for SNPs within the loci of interest. GBS data was extracted from chromosomes 1 (4–12 Mb), 2 (7–11 Mb), 4 (60–63 Mb), and 9 (56.4–57.2) using VCFtools package (Danecek *et al*. 2011). Imputation was conducted on each genomic region separately using Beagle 4.1 (Browning and Browning 2016). Pairwise SNP differentiation (*F*_ST_) between the Chinese and SAP lines was calculated, and the outliers loci in the selected genomic regions were detected on the basis of an inferred distribution of neutral *F*_ST_ using the OutFLANK R package (Whitlock *et al*. 2015). Allelic frequency of the first and second-generation KASP markers in the 30 Chinese and 390 SAP lines was calculated on unimputed GBS data from Hu *et al*., (2019). A global set of 1813 geo-referenced lines, a subset of geo-referenced GBS lines (Lasky *et al*. 2015) and 10 K GBS sorghum lines (Hu *et al*. 2019), was used to determine the allelic distribution of first- and second-generation markers. Among the 10 K GBS sorghum lines, 114 US lines were used for determining the allelic distribution. World map with allelic distribution of the georeferenced lines was generated using ggplot2 (Wickham 2016).

### KASP genotyping

Two 6 mm diameter leaf punches were collected from 3-week-old seedlings for KASP genotyping. Leaf tissue samples were dried using silica-gel beads and shipped to Intertek, Sweden, for genotyping. SNPs for the first-generation markers were selected within the QTL mapped using the AB16 manual phenotyping data. Second-generation KASP markers were designed based on the SNPs identified using JLM, from 2016 and 2017 manual phenotyping data, and SNP outliers detected using *F*_ST_ analysis that were in LD with the peak JLM SNPs. KASP markers were designed using Primer3Plus (Untergasser *et al*. 2007) based on the sorghum reference genome v3.1 with settings product size <120 bp, annealing temperature between 55°C and 62°C with optimum at 57°C, and paired primers annealing temperature within 1°C of one another (Supplementary Table 2). Tails for use with the KASP genotyping system (Semagn *et al*. 2014) were added post primer development, with the HEX-fluorescence designated tail added to the CT allele. DNA extraction and KASP genotyping was conducted at Intertek.

### CT breeding populations

KASP (LGC Biosearch Technologies, Middlesex, UK) genotyping markers were tested on breeding populations from 2 independent US public sorghum breeding programs, the United States Department of Agriculture-Agricultural Research Service (USDA-ARS) Cropping Systems Research Laboratory (CSRL) (Lubbock, Texas) and WKARC sorghum breeding programs. In the USDA-CSRL program, first-generation KASP markers were tested on intercross breeding populations derived from the genetic crosses between 12 US elite grain sorghum lines (BTx399, BTx644, BTx645, BTx2752, B403, BDL0357, BTx642, BTx623, B.10001, B.10004, RTx430, and BTx398*ms3*) and 3 Chinese lines [Hong Ke Zi, Kaoliang, and UI4827 (PI 408816]). Plastic bag emasculations were conducted on the US elite lines to use them as female parents for generating intercross breeding populations. First-generation markers KASP genotyping results from 2 breeding populations, [BTx642 × (B403 × Hong Ke Zi)] and [BTx642 × (BTx398*ms3* × (BTx623 × Kaoliang)-Sel)], were included in the manuscript. Second-generation markers were evaluated on F_2_ plants generated by selfing F_1_ individuals derived from the genetic cross between BTx2752/BTx642 and a NSZ RIL carrying CT alleles on chromosomes 1, 2, and 4. Schematic presentation of the genetic crosses and generation advancements for CT marker-assisted breeding were included in Supplementary Fig. 2.

In the WKARC sorghum breeding program, second-generation KASP markers were tested on 6 populations generated by crossing 3 breeding lines, ARCH 10747-1, ARCH 10747-2, and ARCH 12045, with 2 chilling NAM RILs (14FS205_Kao and 15FS005_NSZ). The ARCH 10747-2 parental line carried both functional tannin genes, while ARCH 10747-1 and ARCH 12045 carried nonfunctional *Tan1* and functional *Tan2*. The chilling NAM 15FS005_NSZ RIL carried CT alleles on chromosomes 1, 2, and 4. RIL 14FS205_Kao carried CT alleles only on chromosomes 1 and 2, a recombination event before the chromosome 4 CT QTL resulted in the loss of this CT allele in this line. Due to this recombination, the Chr4+ allele in 2 populations, 14FS205_Kao × ARCH12045 and 14FS205_Kao × ARCH10747-1, was replaced with no CT allele. KASP-genotyped F_2_ plants were self-pollinated to derive 204 F_3_ families of different CT allele combinations (−/− sib, Chr1+, Chr2+, Chr4+, Chr1+/Chr2+, Chr1+/Chr4+, Chr2+/Chr4+, or Chr1+/Chr2+/Chr4+).

Second-generation KASP markers were further tested on the CT hybrids generated from the KSU sorghum program. Four chilling NAM RILs (14FS125_Kao, 15FS083_NSZ, 15FS679_HKZ, and 14FS273_Kao), carrying different combinations of CT alleles on chromosomes 2 and 4, were backcrossed onto plastic bag emasculated female plants of BTx623 for 3 generations and then selfed for 1 generation (BC_2_F_2_) to obtain NILs with or without CT alleles. Individuals fixed for contrasting CT alleles were identified in the segregating population via KASP markers. CT hybrids were generated by cross-pollinating the BC_2_F_2_ NILs onto 5 nuclear *male sterile3* (*ms3*) US elite lines (RTx2737*ms3*, RTx430*ms3*, RTx436*ms3*, RTx437*ms3*, and SC110*ms3*). Chinese lines Gai Gaoliang (PI 610727), HKZ, NSZ, Kao, and Shan Qui Red (PI 656025) were the chilling-tolerant controls, while BTx623 was the chilling-sensitive control in all early-planted field trials.

## Results

### UAS phenomics validated CT QTL detected with manual SV ratings

**Fig. 1.**
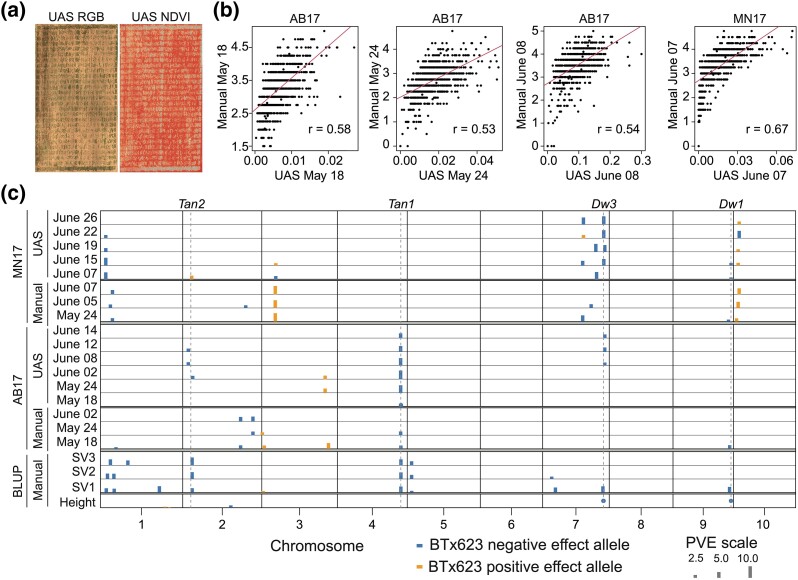
UAS phenomics mapped sorghum CT loci that colocalized with classical tannin genes and dwarfing *Dw3* gene. a) Aerial image of AB17 (Ashland Bottoms 2017) early-planted field trial generated using stitched RGB imagery and NDVI spectrum used for CT phenotyping on June 08, 2017. b) Correlation between manual SV ratings (*y*-axis) and their corresponding date UAS NDVI values (*x*-axis) from 3 AB17 and 1 MN17 (Manhattan 2017) HTP data. Three manual SV rating phenotypes from the AB17 field trial, Manual May 18 (SV1), Manual May 24 (SV2), and Manual June 08 (SV3), were plotted with their corresponding dates UAS May 18, May 24, and June 08 HTP data, respectively. Similarly, Manual June 07 (SV3) data from MN17 was plotted with the UAS June 07 HTP data. c) JLM of UAS NDVI values and manual SV ratings from MN17 and AB17 field trials. JLM of EC and SV rating BLUPs from 6 early-planted field trials and plant height BLUPs from 1 early- and 2 normal-planted field trials. Individual chromosomes were separated by black solid lines and each chromosome was represented based on its genome length. Positive or negative effect of the BTx623 allele was indicated in orange or blue colors, respectively. The percentage of variation (PVE) explained is proportional to the height of the rectangular bar for each locus and loci explaining phenotypic variation >10% were noted with circles. Classical dwarfing and tannin genes were noted with gray dashed lines. RGB, red, green, and blue.

To evaluate the potential of UAS HTP platforms for CT mapping, UAS phenotyping was conducted on 2 sorghum early-planted field trials, AB17 and MN17 (Fig. 1a). Manual phenotyping for early-season seedling performance ratings in AB17 field trial required ∼5 h for each trait. As manual phenotyping was time-consuming, only 3 SV ratings were collected manually for each field trial. In contrast, UAS phenotyping enabled capturing NDVI values several times during the seedling stage. Correlation analysis between AB17 UAS NDVI values and their corresponding 3 manual SV ratings demonstrated an intermediate correlation (0.58, 0.53, and 0.54) (Fig. 1b). Similarly, MN17 NDVI value and its corresponding manual SV rating showed a moderately high correlation (0.67).

JLM of NDVI values from AB17 identified 4 QTL, with each QTL explaining 2.5–7.5% of the phenotypic variation (Fig. 1c). In total, the QTL explained 7–13% variation for the NDVI values (Supplementary Table 3). The chromosome 4 CT QTL was consistently detected across different NDVI values. The QTL on chromosomes 2 and 7 were mapped with 3 NDVI values, and the QTL on chromosome 3 was mapped with 2 NDVI values. Positive alleles were inherited from the Chinese founders, except for the allele on chromosome 3. The QTL on chromosome 4 colocalized (<0.2 Mb) with the classical tannin *Tan1* gene. The QTL on chromosomes 2 and 7 were mapped ∼2 Mb and ∼4 Mb from the classical tannin *Tan2* and the dwarfing *Dw3* gene, respectively. The 4 CT QTL detected with AB17 NDVI values were in proximity to the genomic regions identified using manual SV ratings from best linear unbiased predictions (BLUPs) generated using 6 early-planted field trials. The QTL on chromosome 2, although shifting by a few MB on chromosome 2 with different NDVI dates (Supplementary Table 5), was mapped with NDVI values from a single field season, AB17, while SV rating BLUPs from 6 field seasons were required to detect the chromosome 2 QTL. The observed shift in QTL on chromosome 2 between different NDVI values indicates 2 possibilities, the first one being the same QTL was shifting its coordinates with different NDVI dates and the second possibility of mapping different QTL on chromosome 2 with different NDVI dates.

JLM of MN17 NDVI values mapped 8 QTL, with each QTL explaining 2–7% of the phenotypic variation (Fig. 1c). These QTL in total explained 18–24% variation for NDVI values in MN17 (Supplementary Table 4). The positive alleles on chromosomes 1 and 7 were derived from the Chinese founders. Although the MN17 field trial was planted early, the seedlings emerged under optimal conditions and experienced chilling stress 1 week later. Due to this year-to-year environmental variation, the QTL mapped with MN17 NDVI values were different from AB17 QTL, except for the chromosome 7 QTL mapping close to the *Dw3* gene. The MN17 NDVI QTL on chromosomes 1 and 7 colocalized with the mapped loci of manual BLUPs SV1 rating. The QTL on chromosome 7, close to the *Dw3* gene, was mapped with NDVI values from 1 month after seedling emergence in both AB17 and MN17 field trials. Overall, the QTL mapped with NDVI values were in proximity to the BLUPs manual SV rating QTL reported in Marla *et al*. (2019). Due to fluctuating natural chilling stress between locations and years, the effect size of CT QTL was reduced (Fig. 1c). However, these small detectable QTL effects may directly translate into improved chilling fitness in target environments (Cobb *et al*. 2018).

### Two of the 4 first-generation markers functioned in diverse genetic backgrounds

**Fig. 2.**
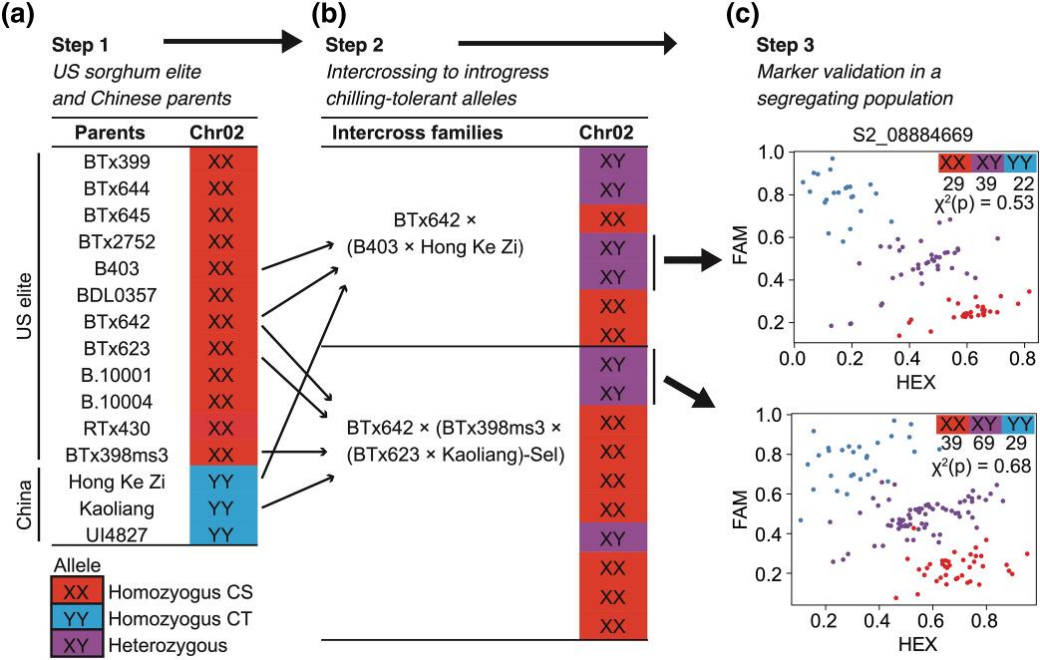
First-generation marker differentiated the US vs Chinese allele in the USDA-CSRL sorghum breeding program. a) Step 1: First-generation marker, designed based on the CT QTL peak SNP on chromosome 2 (S2_08884669), identified the presence of donor allele in the Chinese parents and the alternate allele in the US elite parents. b) Step 2: Intercrosses were conducted between the US and Chinese parents to introgress CT alleles into the US lines. Arrows indicate the parents used for each intercross. Two intercross populations [BTx642 × (B403 × Hong Ke Zi)] and [BTx642 × (BTx398*ms3* × (BTx623 × Kaoliang)-Sel)] genotyped with S2_08884669 marker showed individual plants carrying either homozygous alternate allele (XX) or heterozygous for the donor and the alternate allele (XY). c) Step 3: The progeny of 2 selfed heterozygous (XY) plants segregated in an expected ratio of 1:2:1 for XX:XY:YY (χ2 *P-*values 0.53 and 0.68). Arrows indicate the individual plants that were selfed to generate each intercross population. CT and CS alleles were differentiated through the competitive binding of 2 allele-specific forward primers. The forward primers contained a unique tail sequence that corresponded with 2 universal FRET (fluorescence resonant energy transfer) cassettes, one labeled with FAM dye and the other with HEX dye.

Composite interval mapping of SV ratings from AB16 mapped CT QTL on chromosomes 1, 2, 4, and 9 (Supplementary Table 6). Four KASP genotyping markers (S1_08641374, S2_08884669, S4_61442862, and S9_56611539), generated using the peak QTL SNPs, were developed for marker-assisted breeding. These markers were effective in differentiating the Chinese founders-derived CT allele from the US-derived alternate BTx623 allele. In the USDA-CSRL sorghum breeding program, the KASP marker on chromosome 2 (S2_08884669) revealed the presence of CS allele in 12 elite US sorghum lines and CT allele in 3 Chinese lines (Fig. 2a). The intercross between BTx642 and (B403 × HKZ) F_1_ progeny [BTx642 × (B403 × HKZ)] generated plants with CS/CS or CS/CT alleles in a 1:1 expected ratio (Fig. 2b). Self-pollinating heterozygous plants generated progeny that segregated in a expected ratio of 1: 2: 1 (χ^2^*P*-value 0.53) (Fig. 2c). In the second population, BTx642/(BTx398*ms3*/(BTx623/Kaoliang)- Sel) intercross progeny contained individual plants with CS/CS or CS/CT alleles. Self-pollinated heterozygous (CS/CT) plants generated progeny that segregated in an expected ratio of 1: 2: 1 (χ^2^*P*-value 0.68). Similar to S2_08884669, marker S1_08641374 effectively differentiated the CT allele from the CS allele in 2 segregating populations generated by selfing 2 randomly selected individual plants for desirable traits (Supplementary Figs. 3a–c).

**Fig. 3.**
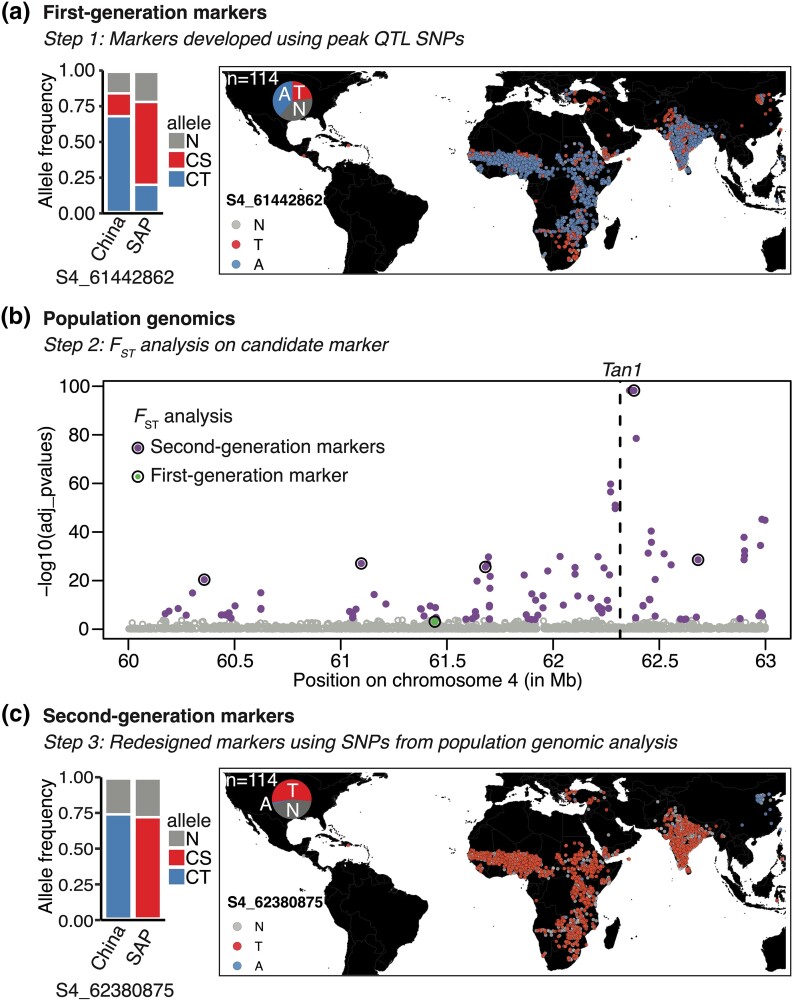
Population genomics enabled detection of polymorphic SNP alleles common in Chinese lines but rare in the global germplasm. a) Allele frequency of the first-generation SNP marker S4_61442862 in 30 Chinese and 390 SAP lines. Global allelic distribution of the CT allele (A) vs the alternate allele (T), and missing genotype information (N) in 1813 georeferenced sorghum lines, which did not contain any US lines. As the 114 US lines in the sorghum 10 K lines do not have georeferences, they were presented as a pie chart. b) *F*_ST_ analysis conducted on 60–63 Mb region of chromosome 4 using the R OutFLANK package. Outlier loci in the selected genomic regions were highlighted in purple, first-generation marker was colored in green, and second-generation markers from the outlier SNPs were highlighted with a circle. The *Tan1* gene at 62.3 Mb on chromosome 4 was noted with a black dashed line. c) Allele frequency of the second-generation SNP S4_62380875 in 30 Chinese and 390 SAP lines, and its global allelic distribution in 1813 georeferenced sorghum lines.

By contrast, first-generation markers S4_61442862 and S9_56611539 failed to differentiate the US vs Chinese parents (Supplementary Figs. 3d and 1e). Allelic distribution of the 4 KASP SNP markers showed missing (N) allelic information in 20–60% of the 30 Chinese and 390 SAP lines (Supplementary Fig. 4 and Fig. 3a). The CT allele for SNPs S1_08641374, S2_08884669, and S9_56611539 was the only detected allele in the Chinese lines (Supplementary Fig. 4). In the SAP, CT alleles for S1_08641374, S2_08884669, and S9_56611539 were observed at 12, 16, and 6% frequency, respectively. In the Chinese lines, the CT allele for S4_61442862 was observed at 68% and the CS allele at 16% frequency. In the SAP, the CT allele S4_61442862 was detected in 20% of the lines. Allelic distribution of S4_61442862 in 1813 georeferenced sorghum lines, a subset of the 10 K GBS lines (Hu *et al*. 2019), revealed the CT allele was a common allele in the global germplasm (Fig. 3a). In summary, the first-generation markers designed to introgress CT alleles were designed based on polymorphisms that were common alleles in the global germplasm.

### Population genomics analysis enabled identification of second-generation markers

Second-generation KASP markers were designed based on the high-resolution mapping loci detected using JLM analysis with 43 K SNPs and 771 RILs (Marla *et al*. 2019). Given that the first-generation markers were designed using globally common CT alleles, *F*_ST_ test was conducted on SNPs within the loci of interest to identify alleles common in the Chinese germplasm but were rare in the global germplasm. For e.g. *F*_ST_ analysis of 60–63 Mb region on chromosome 4 with 30 Chinese and 390 SAP lines revealed an average *F*_ST_ of 0.11. Based on the Bonferroni-adjusted *P*-value < 0.01, *F*_ST_ analysis identified 128 outlier SNPs in the genomic regions of interest (Fig. 3b). The most extreme *F*_ST_ outliers on chromosome 4 colocalized with the *Tan1* gene.

Given that first-generation marker S4_61442862 was not in LD with the most extreme FST outlier on chromosome 4 (Supplementary Table 7), additional FST outlier SNPs were used to develop second-generation KASP markers that were flanking the first-generation marker. Five KASP markers, 1 at the JLM QTL peak (S4_62380875) and 4 flanking markers (S4_60623655, S4_61096729, S4_61680898, and S4_62682585) were developed to introgress the CT allele into chilling-sensitive US sorghums (Supplementary Table 2). Among these markers, CT allele was the common allele in the Chinese lines while being rare in the SAP (Fig. 3c and Supplementary Fig. 5a). Allelic distribution of the peak *F*_ST_ outlier SNP (S4_62380875) in 1,813 georeferenced lines revealed the second-generation marker CT allele was the common allele in Chinese lines but rare in the global germplasm (Fig. 3c). Similar to chromosome 4 QTL, *F*_ST_ analysis was used to develop second-generation KASP markers to introgress CT loci mapped on chromosomes 1, 2, and 9 (Supplementary Figs. 5b, 6a, and 6c). Allelic frequency of the markers on chromosomes 1, 2, and 9 revealed CT allele was the common allele in the Chinese lines while being rare in the SAP (Supplementary Figs. 5c, 6b, and 6d).

### Second-generation markers accurately detected the CT allele in diverse USDA-CSRL breeding lines

**Fig. 4.**
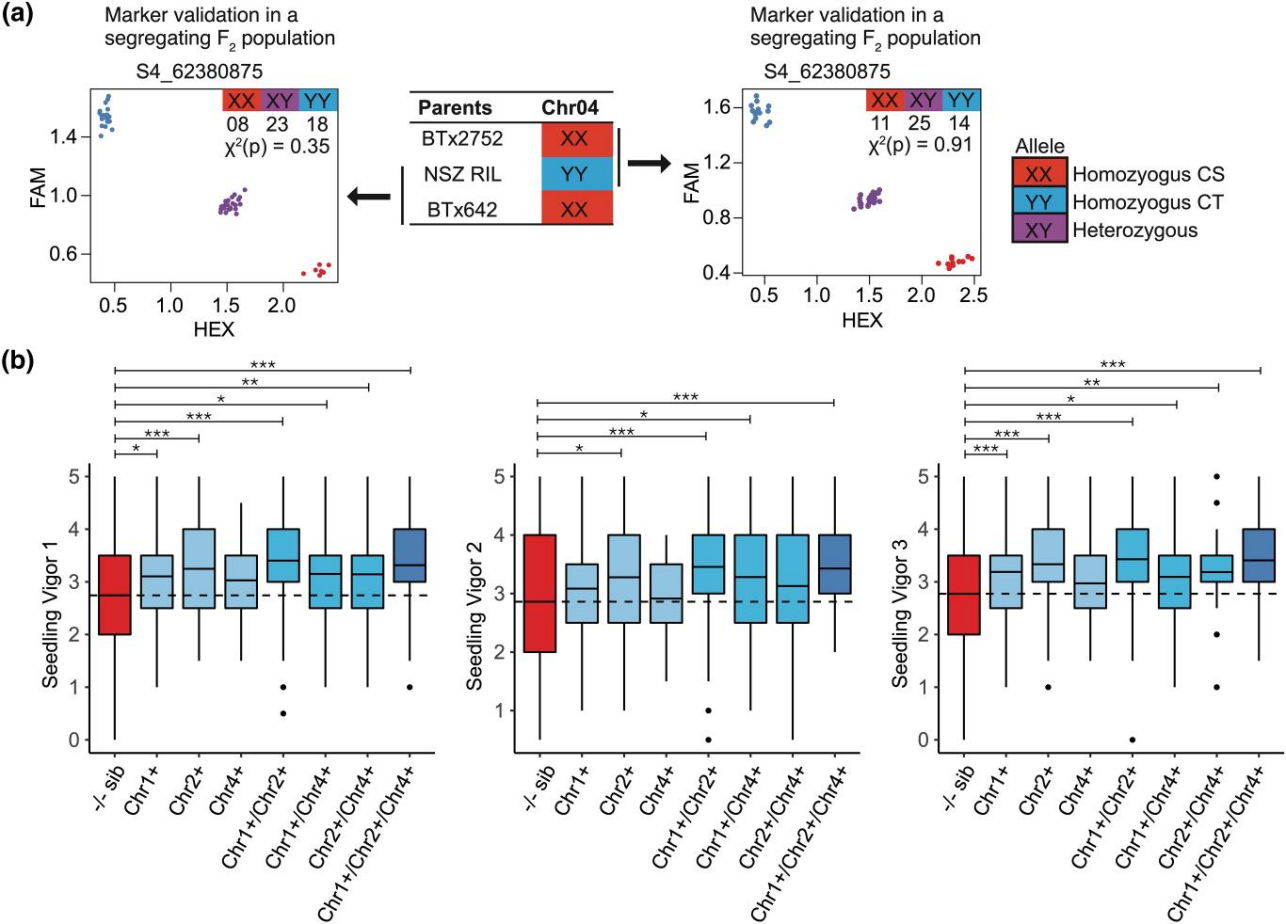
Testing of segregants in independent sorghum breeding programs validated the utility of second-generation markers in improving early-season CT. a) Second-generation marker S4_62380875, generated using outlier *F*_ST_ SNPs, identified the presence of donor allele in the BTx623 by NSZ RIL and the alternate allele in 2 US elite lines. Two F_2_ populations, generated by selfing the F_1_ progeny of BTx642 × NSZ RIL and BTx2752 × NSZ RIL, segregated in an expected ratio of 1:2:1 for XX:XY:YY (χ^2^*P*-values 0.42 and 0.91). CT and CS alleles were differentiated through the competitive binding of 2 allele-specific forward primers containing the FAM dye or the HEX dye. b) Second-generation markers were used to identify the F_2_ plants with different CT allele combinations from a segregating population generated by crossing 3 WKARC lines with 2 chilling NAM RILs. The F_3_ families with different CT alleles were screened for their early-season chilling response under natural chilling stress in Hays 2019. In the F_3_ families, control −/− sib has no CT alleles, single CT allele families carried Chr1+, Chr2+, or Chr4+ from chromosomes 1, 2, or 4, 2 CT alleles stacked families carried Chr1+ Chr2+, Chr1+ Chr4+, or Chr2+ Chr4+ allele combinations, and the 3 CT alleles stacked family carried Chr1+ Chr2+ Chr4+. Three SV ratings (SV1–3) from weeks 1, 2, and 4 after emergence were included. The mean of each F_3_ family was represented as a black horizontal line in the box plots. Lines above box plots indicate pairwise comparisons with a statistically significant differences with *P*-value was less than 0.05 (*), 0.01 (**), and 0.001 (***).NSZ, Niu Sheng Zui.

Second-generation markers, developed using CT alleles that were globally rare but common to locally adapted Chinese germplasm, were effective in differentiating the NSZ × BTx623 RIL CT allele vs the alternate allele in chilling-sensitive US elite lines from the USDA-CSRL breeding program (Fig. 4a). The F_2_ population generated by selfing the progeny derived from the genetic cross between BTx2752 and NSZ RIL, carrying Chr1+, Chr2+, and Chr4+ CT alleles, segregated in an expected ratio of 1: 2: 1 (χ^2^*P*-value = 0.91) with marker S4_62380875. In a second F_2_ population generated from crossing BTx642 with the NSZ RIL, we observed a segregation ratio of 1: 2: 1 ratio (χ^2^*P*-value = 0.42) with S4_62380875. Similar to S4_62380875, second-generation markers on chromosomes 1 and 2 (S1_07620913 and S2_07404837) segregated these 2 F_2_ populations in an expected ratio of 1: 2: 1 ratio (Supplementary Fig. 7). Despite generating chromosome 9 markers, this locus was not used in CT breeding due to its colocalization with the undesirable tall plant *Dw3* allele.

### CT alleles improved early-planted SV in the WKARC sorghum program

To evaluate if the second-generation markers improved early-season CT in an independent breeding program, these markers were used to genotype a segregating F_2_ population derived from a genetic cross between 3 WKARC breeding lines and 2 CT NAM RILs. Under natural chilling stress field trials, the F_3_ family with CS alleles at 1, 2, and 4 CT loci (No CT allele, −/− sib) contained lower average SV (SV1–3) ratings compared to the F_3_ families with CT alleles (Fig. 4b). The F_3_ family with chromosome 2 CT loci (Chr2+) showed 14–20% increase in average SV1–3 ratings compared to the −/− sib control. The F_3_ family with chromosome 1 CT loci (Chr1+) showed 13 and 15% increased SV1 and SV3 ratings, respectively, compared to the −/− sib control. The F_3_ family with chromosome 4 CT allele (Chr4+) showed no significant differences (*P*-value > 0.05) in SV1–3 ratings compared to the −/− sib family. The F_3_ family carrying 2 CT alleles (Chr1+ Chr2+ or Chr1+ Chr4+) showed a significant 12–24% increase in SV1–3 ratings compared to −/− sib control, while the family with Chr2+ Chr4+ showed 14% improved SV1 and SV3 ratings. Stacking all 3 CT alleles (Chr1+ Chr2+ Chr4+) together provided 19–23% higher SV1–3 ratings, compared to the −/− sib control. Taken together, these results demonstrated a significant (*P*-value < 0.05) increase in SV ratings, under natural chilling stress, in the F_3_ families with CT alleles at chromosome 1, 2, and 4 CT loci.

### Marker-assisted breeding increased early-planted SV in US sorghum hybrids

**Fig. 5.**
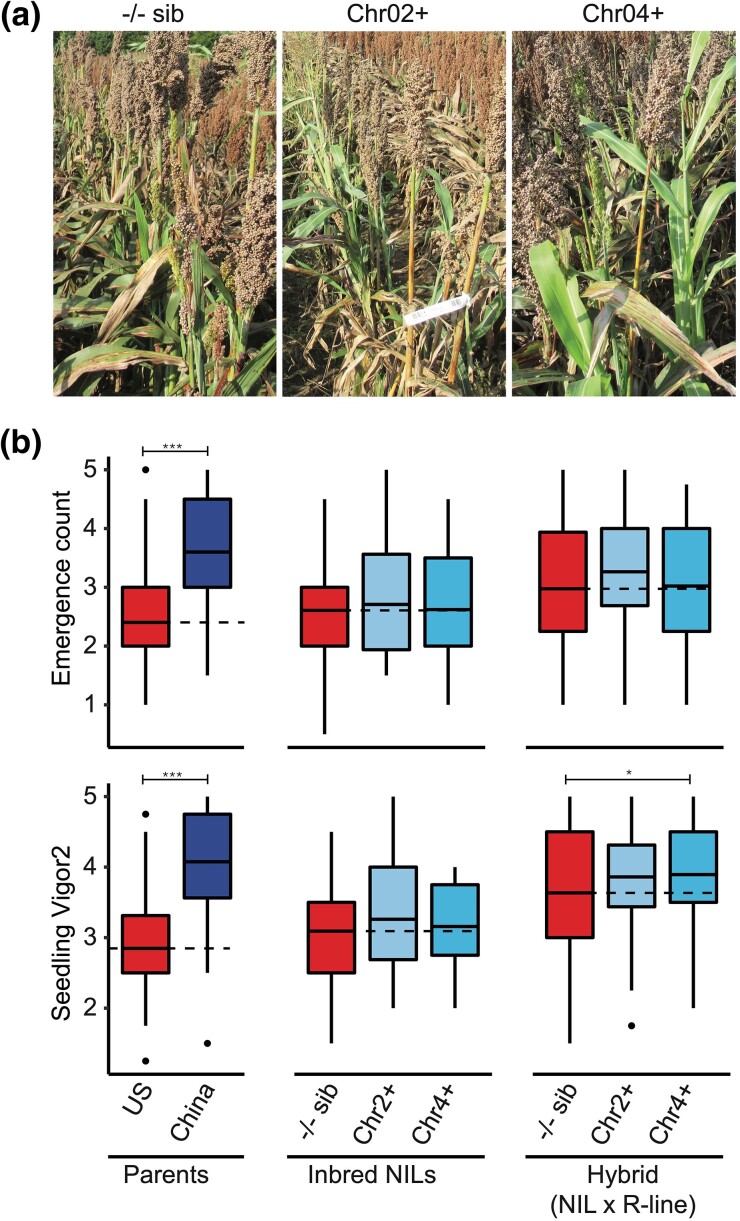
Marker-assisted breeding for CT increased early-planted seedling performance trait ratings in sorghum hybrids. a) Representative field images of sorghum hybrids with Chr2+ or Chr4+ CT alleles and their control −/− sib not carrying any CT alleles. b) Comparison of CT alleles performance in inbred NILs and hybrids carrying no CT allele (−/− sib), Chr2+, or Chr4+ CT allele. Hybrids were generated by crossing 5 US elite R lines carrying the *male sterility3* (*ms3*) gene with inbred NILs. The US and Chinese parents were included as controls. EC and SV2 showed an increase in inbred NILs and hybrids with CT QTL compared to −/− sib. In hybrids, only SV2 contained a significant increase compared to the −/− sib control.

In early-planted field trials, sorghum hybrids developed by crossing inbred NILs, with CT alleles at chr2+, chr4+, or −/− sib, with 5 nuclear *male-sterile3* (*ms3*) elite parents contained similar agronomic characteristics as the US elite inbreds at maturity (Fig. 5a). As expected, early-planted seedling performance traits (EC, SV1, and SV2) were significantly higher (40–50%, *P*-value < 0.001) in the Chinese parents compared to the US elite parents (Fig. 5b and Supplementary Fig. 8), validating the phenotyping experiments. Among the inbred NILs, average EC, SV1 and SV2 ratings were higher in inbreds with Chr2+ or Chr4+ compared to −/− sib. However no significant statistical differences (*P*-value > 0.05) were observed between inbred NILs. In the hybrids, no significant difference was observed with EC between −/− sib, chr2+, or chr4+ hybrids (Fig. 5b). Similarly, no significant differences were observed with SV1 in the hybrids (Supplementary Fig. 8). Significant increase in SV2 (14%, *P*-value = 0.04) was observed only between hybrids carrying chr4+ and the −/− sib, while no significant difference was present between chr2+ and −/− sib hybrids (Fig. 5b).

## Discussion

### UAS phenomics-enabled prebreeding for a complex adaptive trait

Breeding for abiotic stress tolerance was slowed down by the complex genetic architecture of stress tolerance, e.g. sorghum CT (Fig. 1c), and lack of uniform stress under target environments between years and locations (Collins *et al*. 2008; Vandenbroucke and Metzlaff 2013). Additionally, complex trait breeding was impeded by poor scalability due to the time-consuming and tedious nature of manual phenotyping (Araus *et al*. 2018). For example, improving scalability for sorghum CT was limited by the amount of time (∼5 h) required for manually phenotyping each seedling performance trait in a field trial. In this study, UAS phenotyping required 45 min of UAS image capture and NDVI values were extracted in ∼2 h for each trait. JLM with UAS NDVI values mapped CT loci that colocalized with BLUPs manual SV CT loci (Fig. 1c), validating the utility of UAS phenomics in mapping the genetic architecture of complex adaptation traits. UAS phenomics from AB17 field season mapped chromosome 2 QTL, although the QTL position was shifting by a few MB with different traits, while BLUPs from 6 field seasons were required to map the chromosome 2 QTL (Fig. 1c), suggesting increased power of UAS phenomics in complex trait mapping.

For improving the moderate correlation between HTP vs manual data (Fig. 1b), increasing mapping resolution with HTP, and addressing the issue of shifting QTL coordinates/different QTL with different HTP dates in a location, for e.g. AB17 CT QTL on chromosome 2 (Fig. 1c and Supplementary Table 5), possible solutions include reducing UAS height to capture higher resolution images and utilizing improved sensors to quantify small differences in SV. Additionally, manual SV was scored independent of the number of seedlings in each row, while HTP captures NDVI on the entire plot irrespective of the number of plants. Normalizing NDVI data by counting the number of seedlings with HTP and generating the NDVI values based on plant numbers could improve HTP of seedling traits. Given the ease of UAS image acquisition and mapping common CT QTL with UAS and manual phenotyping, improved scalability with UAS phenomics could lead to strong QTL-to-marker associations in applied breeding programs for developing climate-resilient crops (Varshney *et al*. 2021). Application of UAS phenomics was limited to upstream genetic studies for high heritability traits, such as plant height, lodging, and disease resistance (Wang *et al*. 2018; Singh *et al*. 2019; Sarkar *et al*. 2020; Zhou *et al*. 2020; Zhang *et al*. 2021). Our study directly demonstrates the utility of UAS phenomics for prebreeding of complex traits.

### Population genomics generated markers for improved trait predictability

Advances in genomics and quantitative genetics enabled identification of thousands of QTL in public breeding programs, however, most have not yet been used in molecular breeding (Bernardo 2016; Mace *et al*. 2019). Weak trait-to-marker associations, due to low-resolution mapping with bi-parental families, low marker density, and complex LD in the public breeding programs, limited the utilization of mapped QTL in developing improved varieties (Cobb *et al*. 2018). In this study, utilizing a chilling NAM population with 43 K GBS SNPs (Marla *et al*. 2019) and HTP provided high-resolution CT mapping (Fig. 1c) addressed weak trait-to-QTL association from previous sorghum CT studies (Knoll *et al*. 2007; Burow *et al*. 2010). For successful implementation of a public marker development program, the markers developed should function across elite breeding lines from different breeding programs (Bernardo 2016; Cobb *et al*. 2018). Two of the 4 first-generation markers failed to differentiate the Chinese vs US elite lines in the USDA-CSRL program (Supplementary Fig. 3d) as the CT allele was commonly present in diverse sorghum lines (Fig. 3a), indicating the need to develop markers with improved QTL-to-marker association to accurately identify the target allele across diverse genetic backgrounds.

Leveraging population genomic approaches to identify the genomic regions selected in a locally-adapted germplasm and utilizing the polymorphic alleles common in local germplasm but rare globally has the potential to improve marker functionality in diverse breeding programs (Muleta *et al*. 2019). Second-generation KASP markers, developed based on *F*_ST_ analysis outlier SNPs, differentiated the Chinese vs US elite lines in the USDA-CSRL program (Figs. 3c and 4a and Supplementary Fig. 7), indicating the potential of these markers to function in independent public sorghum breeding programs. Although first-generation markers were designed using single-family mapping QTL SNPs and without prior *F*_ST_ analysis, 2 of the 4 markers differentiated the Chinese vs US elite lines in the USDA-CSRL program because the 2 SNPs used for marker development were *F*_ST_ outliers (Supplementary Figs. 5 and 6).

Overall, integrating population genomic analysis in marker development for sorghum CT improved QTL-to-marker association, critical for markers to function in different public marker-assisted breeding programs (Cobb *et al*. 2018). Demonstrating strong marker-to-trait association with second-generation markers, marker-assisted selection improved early-planting SV ratings in the WKARC sorghum breeding program and SV2 in sorghum hybrids with Chr4+ allele in the KSU sorghum breeding program (Figs. 4b and 5b). Considering that chilling stress was not uniform between years and locations, molecular markers detecting CT alleles were developed as a proxy for CT field trials in CT breeding. Increased seedling CT in early-planted field trials showed population genomics-assisted markers were effective in predicting the CT QTL during marker-assisted breeding.

### Combining genomics and phenomics for complex adaptive trait breeding

**Fig. 6.**
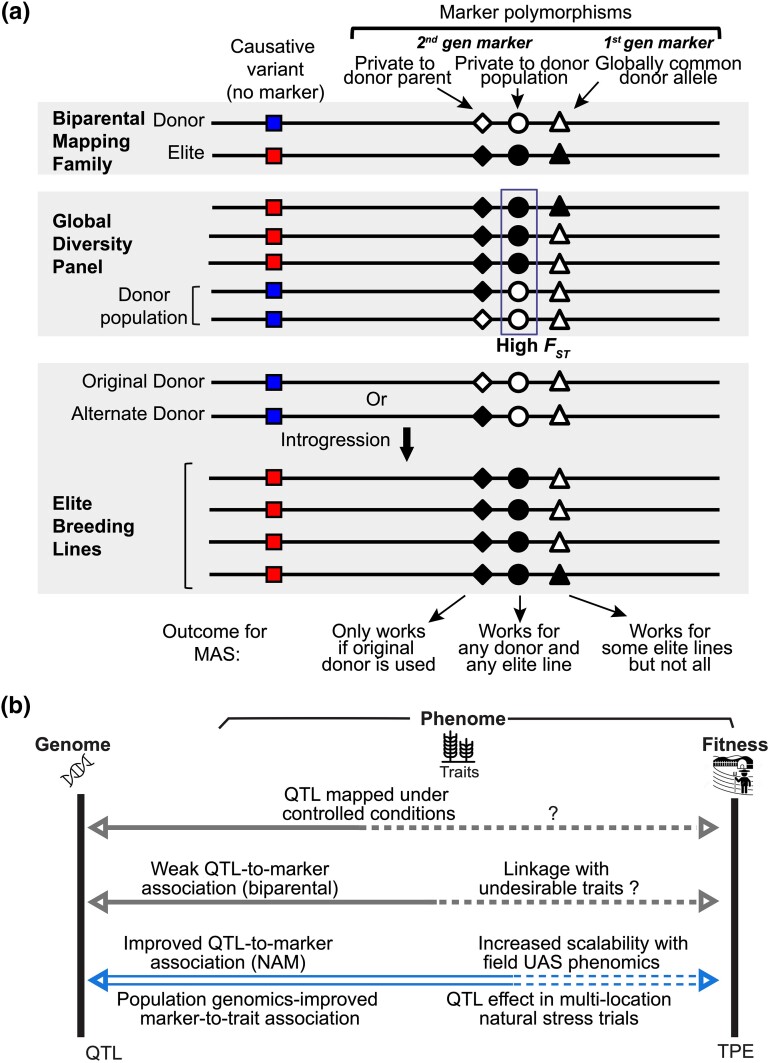
Integrating genomics and phenomics for prebreeding of complex adaptive traits. a) Schematic representation of marker-assisted breeding for introgressing CT alleles into diverse breeding programs. Top panel depicts the allelic distribution of 3 markers, which are in LD with the causative variant, in a biparental mapping family. Panels 2 and 3 represent the allelic distribution of markers in a global diversity panel and elite breeding lines, respectively. All 3 markers will function effectively with the mapping population in which the QTL was detected; however, functionality of these markers in diverse breeding programs depends on the SNP polymorphism commonality in global germplasm. First-generation markers function with the biparental mapping family but fail in different breeding programs with diverse lines as the CT allele is common in global germplasm. Molecular markers generated to identify a private parent allele function only when the same donor parent is used for trait introgression into different breeding programs. Second-generation markers, designed on population genomics outlier SNP alleles conserved in the donor population but rare globally, can function in global diversity panels and elite breeding lines. Second-generation markers private to the donor population can be effective in introgressing desirable alleles into different breeding programs. b) G2P model depicting for complex adaptive trait improvement. Previous breeding efforts for complex trait failed due to failure of QTL mapped in controlled conditions failing to function under TPEs and weak QTL-to-marker associations due to biparental family low-resolution mapping (indicated in gray arrows). Integrating new G2P approaches, that provide improved scalability, QTL-to-marker association, and marker-to-trait association, and the introgressed alleles functioning under TPE in marker-assisted breeding, for marker-assisted breeding of complex adaptive traits under TPE could lead to development of improved climate-resilient crops.

Despite profound knowledge on the genetic architecture of complex traits, limited success stories exist on molecular breeding for complex traits due to the failure of genetics under controlled conditions translating to target population of environments (TPEs), weak trait-to-marker associations, and the functioning of markers and QTL in diverse breeding programs (Blum 2014; Cobb *et al*. 2018; Simmons *et al*. 2021; Ruiz-Vera *et al*. 2022). This sorghum CT presents a case study demonstrating the utilization of G2P approaches for complex trait improvement (Figs. 6a and 6b). Conducting sorghum CT research in natural chilling stress field trials (Figs. 1a and 6b), to replicate relevant spatial and temporal stress variation endured in target environments, addressed the possible failure of controlled conditions genetics translating to TPE in previous studies (Simmons *et al*. 2021; Ruiz-Vera *et al*. 2022).

High-resolution mapping, obtained with the chilling NAM population and high marker density, resolved the earlier problem of weak trait-to-marker associations (Burow *et al*. 2010; Bouchet *et al*. 2017; Marla *et al*. 2019). Markers developed using the donor parent private allele can differentiate the rare vs alternate alleles in a breeding program (Fig. 6a), however, rare allele-specific markers only function when the same private donor parent was used for trait improvement across breeding programs (Cobb *et al*. 2018). Second-generation markers developed using private alleles to the donor population (Figs. 3c and 6a) were effective in detecting polymorphisms in the 2 independent sorghum programs (Fig. 4a and Supplementary Fig. 7), suggesting these markers can be used for marker-assisted breeding in different public CT sorghum breeding programs.

Despite marker-assisted breeding precisely introgressing target loci into elite lines, breeding efforts for complex trait improvement may not be successful due to the failure of introgressed QTL to provide the desired trait in new genetic backgrounds (Cobb *et al*. 2018). Improved seedling CT traits in the WKARC breeding program demonstrated the introgressed CT allele to function in a different genetic background than the mapping population (Fig. 4b). Additionally, improved SV2 rating in the testcross hybrids with Chr4+ CT allele (Fig. 5b), compared to the −/− sib hybrid, indicating that the CT allele functions in sorghum hybrids, commonly used by private commercial US grain sorghum companies (Pfeiffer *et al*. 2019). Overall, leveraging G2P approaches for sorghum early-season CT addressed previous limitations of low-resolution mapping of complex trait genetic architecture, weak trait-to-marker associations, and markers failing to function in independent public breeding programs (Cobb *et al*. 2018; Varshney *et al*. 2021). Our findings from sorghum CT research and development indicate G2P approaches have the potential to successfully work with other complex adaptation traits in different crops (Bernardo 2008; Bohra *et al*. 2020).

## Supplementary Material

jkad116_Supplementary_Data

## Data Availability

GBS data of the chilling NAM population, used for JLM analysis, was previously published in https://doi.org/10.25387/g3.9755336 (Marla *et al*. 2019). Seedling vigor phenotyping from Ashland Bottoms 2019 (AB19) and Hays 2019 (HA19) were included in Supplementary information. Supplementary File 1 contains averaged UAS HTP NDVI values of 2 replicates from the Ashland Bottoms 2017 (AB17) field trial. Supplementary File 2 contains averaged UAS HTP NDVI values of 2 replicates from Manhattan 2017 (MN17) field trial. Supplementary File 3 contains AB17 manual seedling phenotyping data. Supplementary File 4 contains MN17 manual seedling phenotyping data. Supplementary File 5 includes seedling vigor (SV) data from HA19 F3 subpopulations evaluated for their chilling response in the WKARC sorghum breeding program. Supplementary File 6 shows seedling vigor (SV) data from MN19 sorghum hybrids evaluated for their chilling response in the KSU sorghum breeding program. Supplementary File 7 contains chromosome 1 (04–13 MB) hapmap data from 390 sorghum association panel (SAP) and 30 Chinese accessions that were used for FST analysis and to calculate allele frequency of KASP markers. Supplementary File 8 contains chromosome 2 (07–11 MB) hapmap data. Supplementary File 9 contains chromosome 4 (60–63 MB) hapmap data. Supplementary File 10 contains chromosome 9 (56–57 MB) hapmap data. Supplementary material available at figshare: https://doi.org/10.6084/m9.figshare.21358191.
