# Supplementary material for: Genomics and phenomics enabled prebreeding improved early-season chilling tolerance in Sorghum
Source: G3 (Bethesda). 2023 May 26;13(8):jkad116. doi: 10.1093/g3journal/jkad116 (PMC10411554; doi:10.1093/g3journal/jkad116)
Supplement: jkad116_Supplementary_Data [file jkad116_supplementary_data.docx]

# SUPPORTING FIGURES

Supporting material available at Figshare: <https://doi.org/10.6084/m9.figshare.21358191.v2>

Figure S1: Daily minimum air temperature in all early-planted field trials.

Daily minimum temperatures of early-planted field trials from Ashland Bottoms 2016 (AB16_b1 and AB16_b2), Ashland Bottoms 2017 (AB17), Manhattan 2017 (MN17), Hays 2017 (HA17), Hays 2018 (HA18), Hays 2019 (HA19), and Ashland Bottoms 2019 (AB19). Manual seedling data was collected from all locations and UAS high-throughput phenotyping was conducted in AB17 and MN17 field trials. X-axis represents the dates from April 01–June 01 for each location and y-axes represents air temperature in ℃. Sorghum is sensitive to chilling (<15℃), a red dashed line was included for each location to demonstrate chilling stress events during the seedling stage. Blue triangles represent the sowing date and green triangles for seedling emergence.

Figure S2: Schematic of genetic crosses and generation advancement for introgressing CT alleles into diverse sorghum lines.

a) Validation of first-generation markers in diverse sorghum lines, 12 elite sorghum lines and three Chinese lines from the USDA-CSRL sorghum breeding program. Intercross families were generated by crossing Chinese parents with different US sorghum lines. Heterozygous plants identified by KASP genotyping of intercross families with first-generation markers were selfed to generate segregating F2 populations. Two of the four first-generation markers were effective in differentiating CT vs. CS allele in diverse sorghum lines. b) Second-generation markers, developed based on FST outliers, were validated in two F2 populations generated by crossing two elite US lines with a chilling NAM RIL (NSZ RIL). Second-generation markers on chromosomes 1, 2, and 4 differentiated the CT vs. CS allele in both F2 populations. c) Validation of the second-generation markers in the WKARC sorghum breeding program. Three WKARC ARCH lines were crossed with two chilling NAM RILs (containing different combinations of CT QTL) to generate segregating F2 populations. Based on second-generation marker genotyping, 208 F2 individuals with different CT QTL combinations were selfed to generate F3 subpopulations. The F3 subpopulations were screened for their response to chilling stress under early-planted chilling stress in Hays 2019 (HA19). d) CT alleles were introgressed into the US sorghum reference line BTx623 using marker-assisted backcrossing for three generations and then selfed for one generation (BC2F2) to obtain near-isogenic lines (NILs) with or without CT alleles. Pollen from BC2F2 CT NILs was crossed onto five male-sterile3 (ms3) US elite lines to generate sorghum CT hybrids. These hybrids were evaluated for their chilling response in AB19 early-planted field trial.

*Figure S3: First-generation marker genotyping of the US and Chinese lines in the USDA Lubbock sorghum breeding program.*

**a**) Step 1: First-generation marker, designed based on the chilling-tolerant (CT) QTL peak SNP on chromosome 1 (S1_08641374), identified the presence of donor allele in the Chinese lines and the alternate allele in the US germplasm. **b**) Step 2: Intercrosses were conducted between the US and Chinese parents to introgress CT allele into the US lines. Two intercross populations, [BTx642 × (B403 × Hong Ke Zi)] and [BTx642 × (BTx398*ms3* × (BTx623 × Kaoliang)-Sel)] genotyped with S1_08641374 marker showed individual plants carrying either homozygous alternate allele (XX) or heterozygous for the donor and the alternate allele (XY). **c**) Step 3: The segregation pattern of CT/CS alleles observed in the progeny from two randomly selected plants suggest the selfed plants contained XX and XY alleles. Two allele-specific forward primers containing the FAM dye or the HEX dye were CT and CS alleles were used to differentiate the chilling-susceptible vs. the chilling-tolerant allele. **d**) First-generation markers on chromosome 4 and 9 failed to differentiate the US and Chinese parents as the CT allele is common in the global germplasm. **e**) The two intercross populations did not segregate for the marker on chromosome 9 as this population was selected for the *dw1* allele conferring short plant stature.

*Figure S4: Allele frequency of first-generation markers showing the donor allele was common in global diversity.*

Allele frequency of three first-generation markers S1_08641374, S2_08884669, and S9_56611539 was calculated for 30 Chinese and 390 sorghum association panel (SAP) lines separately. Chilling tolerant allele was the only allele identified in the Chinese lines. The presence of CT allele in the SAP indicated the CT allele was not a globally rare allele.

*Figure S5: Population genomics identified markers targeting chilling-tolerant alleles common in locally-adapted lines but rare in global germplasm.*

**a**) Allele frequency of four second-generation markers on chromosome 4, calculated for 30 Chinese and 390 sorghum association panel (SAP) lines separately, showed CT allele was a globally rare allele but a common allele in Chinese lines. **b**) *F*_ST_ analysis conducted on 4–13 Mb region of chromosome 1 using R OutFLANK package. Outlier loci in the selected genomic regions were colored in purple, the first-generation marker in green and highlighted with a circle, and second-generation KASP markers were highlighted with a circle. **c**) Allele frequency of five second-generation markers in 30 Chinese and 390 SAP lines.

*Figure S6: Population genomics-enabled markers identified chilling-tolerant alleles fixed in locally-adapted* lines *but were globally rare.*

**a**) Fixation index (*F*_ST_) analysis of 7–11 Mb on chromosome 2, outlier loci in this region were colored in purple, the first-generation marker indicated in green and highlighted with a circle, and second-generation markers were highlighted with a circle. *Tan2* gene at 7.9 Mb was noted with a black dashed line. **b**) Allele frequency of four second-generation markers on chromosome 2 in 30 Chinese and 390 SAP lines. **c**) *F*_ST_ analysis of 56.4–57.2 Mb on chromosome 9, outlier loci in this region were colored in purple, the first-generation marker indicated in green and highlighted with a circle, and second-generation markers were highlighted with a circle. The tall plant *Dw1* gene at 57 Mb was noted with a black dashed line. **d**) Allele frequency of four second-generation markers on chromosome 9 in 30 Chinese and 390 SAP lines.

*Figure S7: Genotyping of segregants in the USDA-CSRL sorghum breeding program validated the functioning of second-generation markers in an independent breeding program.*

**a**) Second-generation marker S1_11126285 on chromosome 1 was used to genotype two intercross F_2_ populations. The F_2_ populations, generated by selfing the F_1_ progeny of BTx2752 × NSZ RIL and BTx642 × NSZ RIL, segregated in an expected ratio of 1:2:1 for XX:XY:YY (χ2 *p*-values 0.73 and 0.25). **b**) Second-generation marker S2_07404837 on chromosome 2 segregated the F_2_ populations BTx2752 × NSZ RIL and BTx642 × NSZ RIL in an expected ratio of 1:2:1 for XX:XY:YY (χ2 *p*-values 0.83 and 0.08). CT and CS alleles were differentiated through the competitive binding of two allele-specific forward primers containing the FAM dye or the HEX dye. Abbreviations: RIL, recombinant inbred line; NSZ, Niu Sheng Zui; CT, chilling-tolerant; CS, chilling-susceptible.

*Figure S8: Seedling vigor1 comparisons of CT alleles in inbred NILs and hybrids.*

Performance of chilling-tolerant (CT) alleles in inbred near-isogenic lines (NILs) and hybrids carrying Chr2+, Chr4+, or -/- sib (no CT allele). Hybrids were generated by crossing five US elite R lines carrying the *male sterility3* (*ms3*) gene with inbred NILs. The US and Chinese parents were included as controls. Seedling vigor ratings showed an increase in inbreds NILs and hybrids with CT alleles compared to -/- sibs, however, no significant differences were observed between treatments.

# SUPPORTING TABLES

Supporting material available at Figshare: <https://doi.org/10.6084/m9.figshare.21358191.v2>

Table S1: List of Chinese accessions used for FST analysis.

Table S2: KASP genotyping markers used for CT marker-assisted breeding with 100 bp flanking sequences.

Table S3: Joint linkage mapping of NDVI values from AB17 field trial.

Table S4: Joint linkage mapping of NDVI values from MN17 field trial.

Table S5: Linkage disequilibrium analysis of manual SV rating BLUPs and UAS HTP QTL.

Table S6: Composite interval mapping of AB2016 early-planted field trial.

Table S7: Linkage disequilibrium analysis of first- and second-generation KASP markers.
